# Get Smart About Antibiotics Week — November 18–24, 2013

**Published:** 2013-11-15

Antibiotics are an essential tool to treat bacterial infections, but inappropriate use of these drugs has promoted antibiotic resistance and compromised their effectiveness. Health-care providers prescribed an estimated 258 million courses of antibiotics to outpatients in the United States in 2010 (1). Among respiratory conditions for which antibiotics are rarely indicated (e.g., colds and bronchitis), half of ambulatory care visits result in a prescription for antibiotics, most of which are for broad-spectrum antibiotics (2).

In acute care hospitals, each year approximately half of all patients admitted receive an antibiotic, and nearly 50% of antimicrobial use in hospitals is unnecessary or inappropriate (3). Drug-resistant infections are on the rise among hospitalized patients, and options for antibiotic treatment are now severely limited or sometimes nonexistent.

November 18–24, 2013, is Get Smart About Antibiotics Week, an annual observance to coordinate the work of CDC’s Get Smart: Know When Antibiotics Work and Get Smart for Healthcare programs, state-based appropriate antibiotic use campaigns, nonprofit partners, and for-profit partners during a week-long observance on antibiotic resistance and the importance of appropriate antibiotic use. Information on scheduled activities and how to participate during the observance week is available at http://www.cdc.gov/getsmart.
